# Task shifting in primary eye care: how sensitive and specific are common signs and symptoms to predict conditions requiring referral to specialist eye personnel?

**DOI:** 10.1186/1478-4491-12-S1-S3

**Published:** 2014-05-12

**Authors:** Hery Harimanitra Andriamanjato, Wanjiku Mathenge, Khumbo Kalua, Paul Courtright, Susan Lewallen

**Affiliations:** 1 Ministry of Public Health, Antananarivo, Madagascar; 2 Rwanda International Institute of Ophthalmology, Kigali, Rwanda; 3 Blantyre Institute for Community Ophthalmology and Ministry of Health, Lions Sight First Eye Hospital, Blantyre, P.O. Box E180 Post Dot Net, Blantyre, Malawi; 4 Department of Ophthalmology, University of Malawi College of Medicine, P/Bag 360, Blantyre, Malawi; 5 Kilimanjaro Centre for Community Ophthalmology International, Division of Ophthalmology, University of Cape Town, South Africa; 6 University of Cape Town, Department of Ophthalmology, Groote Schuur Hospital, Cape Town, South Africa

**Keywords:** primary eye care, task shifting, blindness, Africa, ocular symptoms, ocular signs, soins de la vue primaires, délégation de tâches, cécité, Afrique, symptômes oculaires, signes oculaires

## Abstract

**Background:**

The inclusion of primary eye care (PEC) in the scope of services provided by general primary health care (PHC) workers is a ‘task shifting’ strategy to help increase access to eye care in Africa. PEC training, in theory, teaches PHC workers to recognize specific symptoms and signs and to treat or refer according to these. We tested the sensitivity of these symptoms and signs at identifying significant eye pathology.

**Methods:**

Specialized eye care personnel in three African countries evaluated specific symptoms and signs, using a torch alone, in patients who presented to eye clinics. Following this, they conducted a more thorough examination necessary to make a definite diagnosis and manage the patient. The sensitivities and specificities of the symptoms and signs for identifying eyes with conditions requiring referral or threatening sight were calculated.

**Results:**

Sensitivities of individual symptoms and signs to detect sight threatening pathology ranged from 6.0% to 55.1%; specificities ranged from 8.6 to 98.9. Using a combination of symptoms or signs increased the sensitivity to 80.8 but specificity was 53.2.

**Conclusions:**

In this study, the sensitivity and specificity of commonly used symptoms and signs were too low to be useful in guiding PHC workers to accurately identify and refer patients with eye complaints. This raises the question of whether this task shifting strategy is likely to contribute to reducing visual loss or to providing an acceptable quality service.

## Abstract (fr)

**Résumé:**

**Contexte**

L’inclusion des soins de la vue primaires dans la liste des services offerts par les fournisseurs de soins de santé primaires s’inscrit dans une stratégie de « délégation de tâches » visant à améliorer l’accès aux soins de la vue en Afrique. En théorie, la formation en soins de la vue primaires enseigne à ces fournisseurs à reconnaître des signes et symptômes précis ainsi qu’à traiter et à aiguiller les cas en fonction de ceux-ci. Nous avons mesuré la sensibilité et la spécificité de ces signes et symptômes permettant de détecter les pathologies oculaires importantes.

**Méthodes**

Des employés spécialisés en soins de la vue de trois pays africains ont évalué, à l’aide d’une lampe, les signes et symptômes particuliers que présentaient les patients qui s’étaient rendus à leur clinique. Ils ont ensuite procédé à un examen plus complet, nécessaire pour établir des diagnostics définitifs et gérer les cas en question. On a ainsi mesuré la sensibilité et la spécificité des signes et symptômes permettant de détecter les pathologies oculaires nécessitant un aiguillage vers un spécialiste ou menaçant la vue du patient.

**Résultats**

La sensibilité permettant de détecter les pathologies menaçant la vue allait de 6,0 % à 55,1 % selon le signe ou le symptôme; la spécificité, quant à elle, allait de 8,6 % à 98,9 % selon le signe ou le symptôme. Si l’on combinait tous les signes et symptômes, la sensibilité montait à 80,8 %, mais la spécificité était de 53,2 %.

Dans le cadre de cette étude, la sensibilité et la spécificité des signes et symptômes courants étaient trop faibles pour être utiles aux fournisseurs de soins de santé primaires, qui doivent détecter avec précision les pathologies oculaires et aiguiller les patients. À la lumière de ces résultats, on peut se demander si la stratégie de délégation de tâches peut aider à réduire l’incidence de la perte de la vue ou à offrir des services d’une qualité acceptable.

## Background

Blindness and visual impairment constitute a public health problem in sub-Saharan African countries. In response to this, and recognizing that about 80% of blindness is avoidable, the World Health Organization (WHO) and the International Agency for the Prevention of Blindness (IAPB) have elaborated and launched “VISION 2020 – The Right to Sight” for the elimination of avoidable blindness by the year 2020 [1]. One of the important constraints to achieving this goal is the shortage of health workers trained in eye care [2]. In an effort to overcome this, primary eye care (PEC) as an integral part of primary health care (PHC) has been recommended as a key strategy. PEC means different things in different parts of the world [3]. In settings with more resources, “primary eye care” refers to services delivered by nurses and health assistants who are trained for and work more or less exclusively in eye care, such as ophthalmic clinical officers or ophthalmic nurses and assistants. These “dedicated eye workers” exist in sub-Saharan Africa, but are usually considered “mid-level” eye care workers, stationed at secondary or even tertiary centers. PEC, in sub-Saharan Africa, is considered to be eye care delivered at the primary health care level, by general PHC workers who are also responsible for immunization, maternal child health, and other primary health care duties [3]. Thus PEC is an example of “task shifting,” i.e., shifting the provision of basic eye services from dedicated eye workers to general health workers at the primary level. One presumed advantage of this is to make eye services more accessible at the community level.

Since managing ocular complaints is a small part of their workload, PHC workers will have limited expertise in eye care. There is evidence that PHC workers sometimes overstep their competence in treating conditions, and ready availability of ocular antibiotics can lead to a false sense of security in “treating” patients with eye problems [4,5]. Easy access to antibiotic eye ointment such as tetracycline was possibly useful in an era when trachomatous conjuctivitis was very common, but trachoma has declined in many parts of Africa [6]. PEC training for PHC workers, although not standardized, usually focuses on the recognition of specific signs and symptoms as triggers for treatment or referral. These are signs and symptoms that are emphasized in most ophthalmology textbooks and that can indicate serious problems when considered in the context of patient history and further examination. Symptoms include complaints of difficulty seeing or pain; signs include reduced visual acuity, redness (hyperemia), lack of corneal clarity, and abnormal appearance of the pupils. Most training in primary eye care emphasizes the importance of some or all of these and they are presumed to be relatively easy to evaluate by history taking and examination with a torch.

To our knowledge, the sensitivity and specificity of basic symptoms and signs included in PEC training to guide management has not been quantified; but this knowledge would be key to know how well we can expect PHC workers to use these to provide a reasonable standard of eye care. We undertook this study in order to test the sensitivity and specificity of several symptoms and signs when evaluated by experienced eye care providers. We chose to test the value of the signs and symptoms when evaluated by experienced eye care providers rather than by PEC workers in order to test the intrinsic value of the signs and symptoms and not the ability of the PHC workers to evaluate them.

## Methods

The study was conducted prospectively in the context of routine examinations of new patients who presented consecutively to eye clinics, without special referrals. The clinics were in eye departments in general hospitals and all accepted walk-in, self-referred patients; thus they represent patients who present to first line health care workers. The examiners were ophthalmologists and ophthalmic clinical officers employed in 11 eye clinics (5 in Madagascar, 3 in Malawi and 3 in Rwanda). Each examiner was asked to enroll at least 100 new patients sequentially. Standardized forms were used to collect the information.

The visual acuity was measured and recorded on the form before the examination began. The examiner then asked the patient specifically if he had a problem with distance or near vision, pain, discharge or itching in either eye. Then the examiner assessed whether the eyes were red, pupil was black, eyes were straight, and lids closed normally, using only a torch. Examiners were instructed to try to conduct this examination as objectively as possible.

After these data were recorded, the examiner conducted an examination according to his usual practices, using a slit lamp, ophthalmoscope, and dilation of the pupil or whatever else he judged necessary to make a definite diagnosis and manage the patient. After this more thorough examination, he recorded the diagnosis for each eye as normal or selected all that applied of the following 10 diagnoses: cataract, glaucoma, conjunctivitis, keratitis, uveitis, retinal disease, eyelid disease, neuro-ophthalmologic disease, refractive error, and “other”. “Normal” eyes had to have a visual acuity of 6/18 or better. Examiners were instructed not to go back and make any changes in the initial torch exam following their more thorough examination and management decision.

Forms were sent by post to one center for data entry and analysis. Double data entry was performed using Epi Data and data entry errors were reconciled. Then data were imported into Stata 11 for tabulation of frequencies of the signs and symptoms and of final diagnoses; these were used to calculate sensitivity, specificity and predictive values.

We defined “serious” eye diagnoses as those that would require referral to specialist eye care personnel for management. This included all diagnoses except “normal” and conjunctivitis. We calculated sensitivity, specificity, and predictive values (positive and negative) for identifying serious diagnoses for each of the subjective symptoms and the signs recorded from the torch examination. We also combined signs and symptoms into “referral criteria” and tested the sensitivity of these.

Recognizing that there might be other definitions of “serious” in ocular pathology, we calculated sensitivity, specificity, and predictive values of the signs and symptoms to identify sight threatening (ST) and non-sight threatening (NST) conditions, where the latter included diagnoses of normal, conjunctivitis or refractive error (including presbyopia).

## Results

We collected observations on 494, 313, and 490 patients (total = 1297) from Madagascar, Malawi, and Rwanda, respectively. The mean ages were 40.8, 37.4, and 36.9 years; males comprised 42.3%, 42.5%, and 44.4% of the populations, respectively, for Madagascar, Malawi, and Rwanda. The Malagasy patients were more likely to have refractive errors (including presbyopia) than the patients in Malawi and Rwanda but the frequency of other diagnoses was similar. We analysed right and left eyes separately; they were very similar so we report only on right eyes.

Table 1 shows the sensitivity, specificity, and predictive value for individual signs and symptoms. In this population, a complaint of “poor near vision” was the most sensitive sign of a diagnosis requiring specialist care, but sensitivity was only 52.9%. All other signs and symptoms had lower sensitivities, ranging from 4.1% to 47.4%.

**Table 1:** Value of signs and symptoms for differentiating "serious" from "non-serious" eye conditions

	Right eyes						
Sign or symptom	Serious	Not serious	Total	Sensitivity	Specificity	PPV	NPV

*poor distance *good distance *Total*	361 400 *761*	29 449 *478*	*390* *849* *1239*	47.4	93.9	92.6	52.9

*poor near vision *good near vision *Total*	401 357 *758*	45 429 *474*	*446* *786* *1232*	52.9	90.5	89.9	54.6

VA<6/18 VA≥ 6/18 *Total*	288 468 *756*	12 460 *472*	*300* *928* *1228*	38.1	97.5	96.0	49.6

*pain no pain *Total*	276 485 *761*	188 287 *475*	*464* *772* *1236*	36.3	60.4	59.5	37.2

discharge no discharge *Total*	96 661 *757*	88 386 *474*	*184* *1047* *1231*	12.7	81.4	52.2	36.9

*itching no itching *Total*	190 566 *756*	277 199 *476*	*467* *765* *1232*	25.1	41.8	40.7	26.0

not straight straight *Total*	91 662 *753*	21 454 *475*	*112* *1116* *1228*	12.1	95.6	81.3	40.7

lid closure abnormal lid closure normal *Total*	31 727 *758*	7 466 *473*	*38* *1193* *1231*	4.1	98.5	81.6	39.1

eye red eye not red *Total*	188 568 *756*	136 338 *474*	*324* *906* *1230*	24.9	71.3	58.0	37.3

pupil not black pupil black *Total*	110 645 *755*	1 472 *473*	*111* *1117* *1228*	14.6	99.8	99.1	42.3

Row and column totals are in italics. Data are missing for some eyes. PPV = predictive value of a positive test (abnormal sign). NPV = predictive value of a negative test (absence of the abnormal sign).

*Subjective patient report.

Table 2 illustrates the results when referral criteria #1 (VA<6/18 or pain) and referral criteria #2 (VA<6/18 or pain or red eye) were used. These provided sensitivities of 64.0% and 69.1%, respectively, while specificities were 60.4% and 50.6%.

**Table 2:** Sensitivity, specificity and predictive value for two other referral criteria

	Right eyes						
Referral Criteria	Serious	Not serious	Total	Sensitivity	Specificity	PPV	NPV

VA<6/18 or pain Neither of above *Total*	504 283 *787*	202 308 *510*	*706* *591* *1297*	64.0	60.4	71.4	52.1

VA<6/18 or pain or red eye None of above *Total*	544 243 *787*	252 258 *510*	*796* *501* *1297*	69.1	50.6	68.3	51.5

Row and column totals are in italics. Data are missing for some eyes. PPV = predictive value of a positive test (abnormal sign). NPV = predictive value of a negative test (absence of the abnormal sign).

Table 3 shows the sensitivity, specificity, and predictive values for identifying “sight threatening conditions” as defined in the methods above. A complaint of poor distance vision had the highest sensitivity (51.9%). Table 4 shows the results of using referral criteria # 1 or #2 to identify “sight threatening conditions.” These criteria provided sensitivities of 74.5% and 80.9%, respectively.

**Table 3:** Value of signs and symptoms for differentiating "sight threatening" from "non-sight threatening" eye conditions

	Right eyes						
Sign or symptom	ST	NST	Total	Sensitivity	Specificity	PPV	NPV

*poor distance *good distance *Total*	287 266 *553*	120 622 *742*	*407* *888* *1295*	51.9	83.8	70.5	70.0

*poor near vision *good near vision *Total*	284 266 *550*	167 571 *738*	*451* *837* *1288*	51.6	77.4	63.0	68.2

VA<6/18 VA≥ 6/18 *Total*	305 248 *553*	668 63 *731*	*973* *311* *1284*	55.1	8.6	31.3	20.3

pain no pain *Total*	246 307 *553*	243 496 *739*	*489* *803* *1292*	44.5	67.1	50.3	61.8

discharge no discharge *Total*	95 455 *550*	95 641 *736*	*190* *1096* *1286*	17.3	87.1	50.0	58.5

itching no itching *Total*	165 386 *551*	326 411 *737*	*491* *797* *1288*	29.9	55.8	33.6	51.6

not straight straight *Total*	77 468 *545*	48 691 *739*	*125* *1159* *1284*	14.1	93.5	61.6	59.6

lid closure abnormal lid closure normal *Total*	33 517 *550*	8 729 *737*	*41* *1246* *1287*	6.0	98.9	80.5	58.5

eye red eye not red *Total*	191 358 *549*	154 583 *737*	*345* *941* *1286*	34.8	79.1	55.4	62.0

pupil not black pupil black *Total*	110 437 *547*	3 734 *737*	*113* *1171* *1284*	20.1	99.6	97.3	62.7

Row and column totals are in italics. Data are missing for some eyes. PPV = predictive value of a positive test (abnormal sign). NPV = predictive value of a negative test (absence of the abnormal sign).

*Subjective patient report.

**Table 4:** Sensitivity, specificity and predictive value for two other referral criteria

	Right eyes						
Referral Criteria	ST	NST	Total	Sensitivity	Specificity	PPV	NPV

VA<6/18 or pain Neither of above *Total*	413 141 *554*	293 450 *743*	*706* *591* *1297*	74. 5	60. 5	58.5	76.1

VA<6/18 or pain or red eye None of above *Total*	448 106 *554*	348 395 *743*	*796* *501* *1297*	80.8	53.2	56.3	78.8

Row and column totals are in italics. Data are missing for some eyes. PPV = predictive value of a positive test (abnormal sign). NPV = predictive value of a negative test (absence of the abnormal sign).

There were no significant differences in the sensitivities and specificities among the three countries.

## Discussion

Task shifting from a higher trained to a lesser trained cadre is one suggested solution to the health workforce shortage in Africa. The success of this strategy will depend on many factors including, but not limited to, proper supervision [7] and the existence of proven indicators (tests or criteria) that can be used by health care workers without the benefit of much experience, to make good management decisions. The latter requirement, that there be reliable indicators, is critical in any scheme to shift tasks from highly competent specialist medical personnel with in-depth knowledge to generalist health workers with limited specialist knowledge, experience, and equipment, while avoiding an unacceptable decrease in quality of care. The development of successful algorithms and training for primary health care workers is based on likelihoods that specific, easily identifiable signs (indicators) reflect certain pathophysiologic processes. The existence and identification of such indicators determines to a large extent whether it is reasonable to expect a minimally experienced generalist cadre to manage specialist medical conditions. If such indicators cannot be identified then it may not be reasonable to expect “task shifting” to be successful. Useful indicators need to be simple to use and should have high sensitivity and specificity. Sensitivity and specificity are measures of the accuracy of a diagnostic test to detect a condition and, unlike predictive value, do not depend on the prevalence of the condition in the population [8].

In this study, we considered several symptoms and signs that are indicative of eye health and would likely be included in training of PHC workers to help with decision making. We treated these signs and symptoms as diagnostic tests to see how accurately they predicted whether patients in an eye clinic had “serious” eye conditions requiring referral for specialist care. These signs and symptoms are regularly considered by ophthalmic professionals to evaluate patients, however they are supplemented by a considerable body of additional knowledge of the many and varied pathological conditions that can affect the eye. Furthermore, ophthalmic professionals depend on a number of sophisticated instruments and tests in order to detect redness (inflammation), or a pupil that is not “black,” or eyes that are not straight. The possibility of correctly diagnosing many eye conditions, particularly at an early stage, depends on examination with instruments more complex than a torch. The sensitivity of the single symptoms and signs we tested here, evaluated with a torch alone, even by experienced ophthalmic professionals, was very low. High sensitivity (identifying a high percentage of people who have a problem) is particularly important in order to avoid missing conditions that require further evaluation and treatment.

While combinations of symptoms and signs (referral criteria #1 and #2) improved the sensitivity somewhat, the resulting sensitivities were still unacceptably low. As more symptoms and signs are included in the criteria for referral, the sensitivity will increase further, however the specificity will become negligible, resulting in a situation where nearly all patients would be referred. Not only is this inefficient and counterproductive, but it could undermine any confidence of the community in the PHC workers providing eye care.

It is interesting to note that the sensitivity and specificity of these common symptoms and signs has not been tested before in view of the frequent calls for scaling up PEC in Africa. The sensitivity and specificity for several other ocular tests used by non-ophthalmic health personnel including the red reflex, confrontation visual fields, and penlight test for photophobia to detect “serious” pathology has been measured with various results [9-13]. Recognition of the difficulty in determining when an eye is “red” has required investigators interested in conjunctival hyperemia to develop different grading schemes [14,15].

Experience over time, as well as more sophisticated instruments, informs ophthalmic professionals’ decision making. PHC workers might improve their decision making ability with time, however, considering the wide variety of conditions they are expected to deal with every day (many life threatening), it is unrealistic to expect them to gain a lot of experience with eye care patients. We have previously documented that PHC centers in a study in Tanzania see only three eye patients per month on average [16]. It is higher than this in some settings (unpublished data) but still constitutes a minor part of the PHC workers’ duties. Thus, opportunities to practice skills are very limited and experience will remain low amongst this cadre.

The VISION 2020 initiative focuses on cataract since this is responsible for around 50% of blindness in Africa [17]. While a white pupil is indeed a very specific sign for cataract, this sign only indicates very advanced cataracts, when visual acuity has deteriorated to the level of ability to detect light only; this is below the World Health Organization definition of blindness (less than 3/60). Increasingly, patients want and need surgery well before this stage, but diagnosis at earlier stages requires an ophthalmoscope.

There are several limitations to this study. Calculation of sensitivity and specificity depends on having a gold standard. In this case we assumed that the diagnoses made by a group of experienced ophthalmologists or ophthalmic clinical officer would be correct. We did not, however, measure interobserver agreement in diagnoses among our “gold standards” nor provide strict diagnostic criteria. Some data forms were not filled in completely, as evidenced by the missing data in the Tables. Another limitation is that we determined what would constitute “serious” disease *post hoc*, after data collection but before analysis, partly to decrease the potential for bias among the examiners; however we do not expect either of the two definitions we used to be particularly controversial among ophthalmologists. Finally, the population in this study may not be representative of those patients who would present at PHC centers; on the other hand, since these were all new patients to the eye clinics, and no referrals were required, they may be reasonably representative of problems in the community.

In spite of the limitations, we believe that this work raises important questions about the current concept of PEC as a strategy to achieve VISION 2020 goals in sub-Saharan Africa. More testing of the validity of this concept is required. This includes a number of interrelated issues including testing PEC curricula for their usefulness in informing patient management, testing PHC workers’ ability to reliably detect key symptoms and signs, testing how well PEC management guidelines are adhered to by PHC workers, and exploring community attitudes towards PEC delivered at PHC centers. The second issue is described in a companion paper in this issue.

## Conclusions

The sensitivity of symptoms and signs commonly used to help PHC workers to manage eye disease was unacceptably low in this study. These findings need to be tested in other settings and more rigorously. Case identification and proper referral are pillars of primary care; if tools that allow general PHC workers to provide this are not being used, or do not exist, then we need to reconsider whether primary eye care is better provided by a more specialized cadre. PHC workers could be more usefully trained to focus on eye health education messages and less on clinical diagnosis and management of patients.

## List of abbreviations

DFATD: Foreign Affairs, Trade and Development Canada; GHRI: Global Health Research Initiative; IDRC: International Development Research Centre; NPV: negative predictive value; NST: non-sight threatening; PEC: primary eye care; PHC: primary health care; PPV: positive predictive value; ST: sight threatening; VA: visual acuity

## Competing interests

None of the authors have any competing interests.

## Authors’ contributions

All authors were involved in designing the study. HHM, WM, and KK implemented the study and supervised data collection. SL and PC analysed data. All authors contributed to the final manuscript.

## Declarations

The publication costs associated with this article are funded by Foreign Affairs, Trade and Development Canada and the International Development Research Centre through the Global Health Research Initiative.

This article has been published as part of *Human Resources for Health* Volume 12 Supplement 1, 2014: Addressing the human resources for health crisis through task-shifting and retention: results from the Africa Health Systems Initiative's research component. The full contents of the supplement are available online at http://www.human-resources-health.com/supplements/12/S1
